# DFT and TD‐DFT Studies of D‐π‐A Organic Dye Molecules with Different Spacers for highly Efficient Reliable Dye Sensitized Solar Cells

**DOI:** 10.1002/open.202300307

**Published:** 2024-05-03

**Authors:** Nambury Surendra Babu, Maluak Paul Kuot Malang, Ismail Abubakari

**Affiliations:** ^1^ Computational Quantum Chemistry Lab Department of Chemistry College of Natural and Mathematical Sciences The University of Dodoma Post box: 238 41218 Dodoma Tanzania

**Keywords:** Dye-sensitized solar cell (DSSC), DFT, TD-DFT, Intramolecular charge transfer (ICT), donor-π-spacer-acceptor (D-π-A)

## Abstract

This study focuses on six D‐π‐A systems, utilizing diverse π‐spacers as bridges. Comprehensive analysis through Density Functional Theory (DFT) and Time‐dependent Functional Theory (TD‐DFT) methods at B3LYP using 6‐31G (d.p) basis set explores geometrical, electrical, optical, photovoltaic, and absorption properties. E_HOMO_, E_LUMO_, and energy gap (E_gap_), for all of these dyes have been determined and discussed using ground state optimization. TD‐DFT calculates optical properties, unveiling enhanced excitation energies and HOMO‐LUMO energy levels, indicative of improved electron injection and dye regeneration processes. Examination of energy gap, open‐circuit voltage (VOC), free energy change (ΔGinject), light harvesting efficiency (LHE), and absorption spectra reveals D4 dye′s lower Egap and robust absorption in the visible spectrum. Molecular tailoring emerges as a promising technique for optimizing D‐π‐A sensitizer design, offering potential advancements in DSSCs applications.

## Graphical abstract

This study explores six D‐π‐A systems with varied π‐spacers, employing Density Functional Theory (DFT) and Time‐dependent Functional Theory (TD‐DFT) methods. Analysis includes geometrical, electrical, optical, photovoltaic, and absorption properties. Ground state optimization determines EHOMO, ELUMO, and energy gap (Egap). TD‐DFT reveals enhanced excitation energies and HOMO‐LUMO levels, indicating improved electron processes. Notably, the D4 dye exhibits a lower Egap and robust visible spectrum absorption, suggesting molecular tailoring as a promising approach for DSSCs optimization.

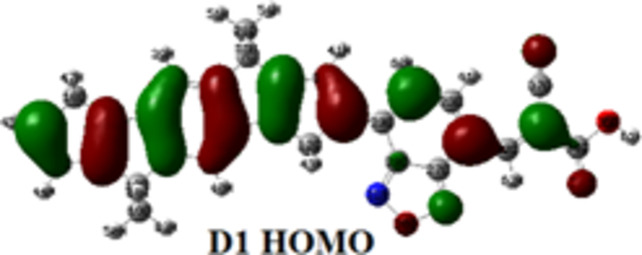

## 1 Introduction

**Figure 1:**
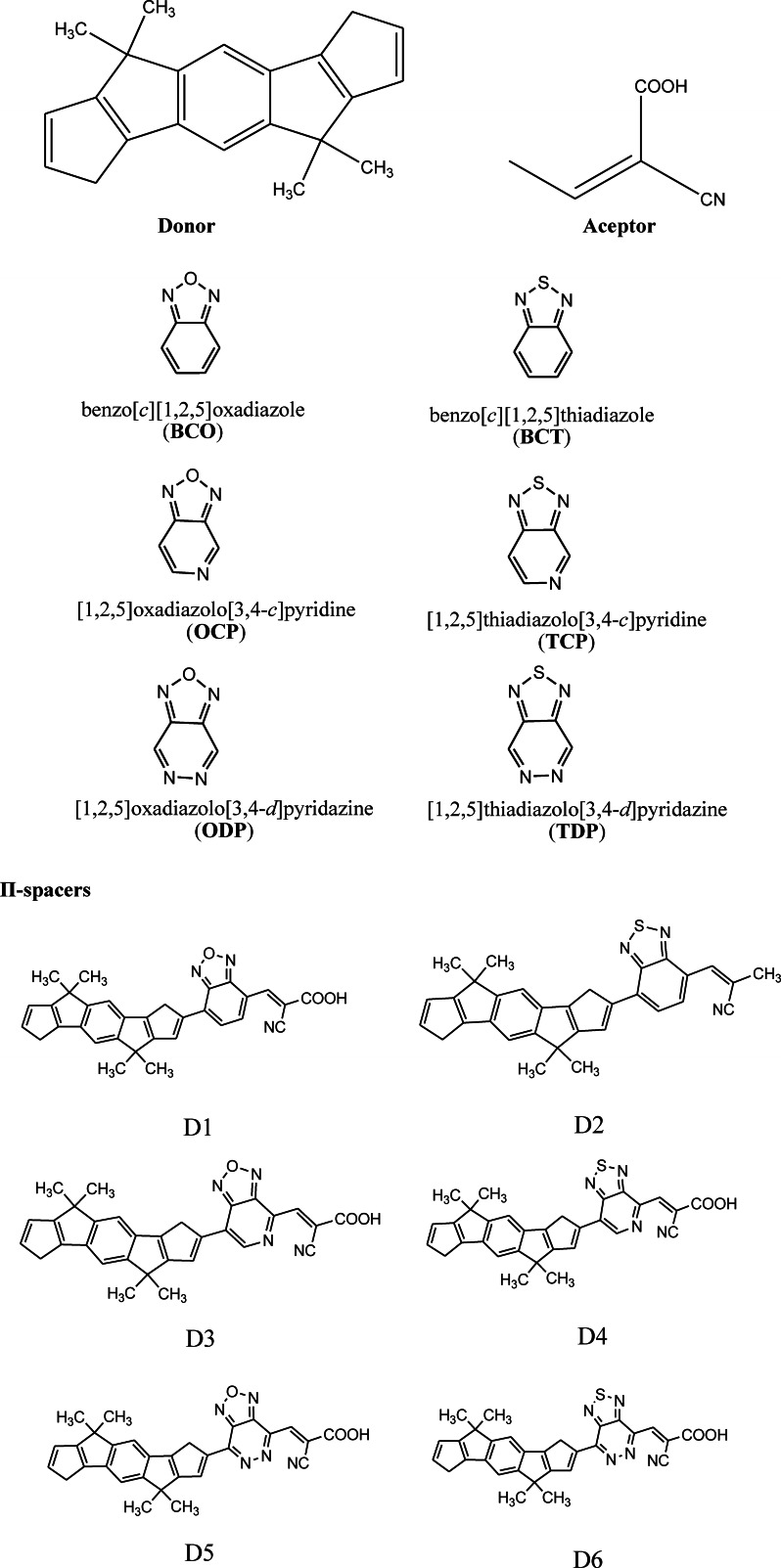
Chemical structure of all dyes under study.

Since conventional energy systems are more energy‐demanding and environmentally hazardous, innovative renewable energy technologies are gaining popularity. Light‐driven separation of charges in molecular structures has attracted a lot of attention in the past few years as a technology for converting and storing solar energy.[^1^, ^2^] With respect to its high‐power conversion, cheap cost, and flexibility, dye‐sensitized solar cells have received considerable interest in scientific and academic studies as well as commercial use since the publication of the first report by O'Regan and Gratzel in 1991.^[3]^ The DSSCS class is comprised of an expanded band gap (TiO2) suitable for collecting light from the visible portions of the spectrum, an electrolyte containing an iodide/tri‐iodide redox couple, and a platinum counter electrode.^[4]^ Photosensitizers are essential for both power conversion efficiency and cell stability. Metal‐free photosensitizers with donor (D), π‐conjugated bridges, and acceptor (A) structures have currently attracted attention as a viable alternative to standard Ruthenium‐based dyes.^[5]^ However, the organic dye has several advantages, including its low cost, ease of synthesis and purification, easy modification of structure, high molar extinction coefficient, and good intermolecular charge transfer (ICT) photoexcitation properties.^[6]^ Many researchers concentrated on their molecular design theoretically and experimentally by changing the donor, π‐spacer, and acceptor units.^[7]^ Triphenylamine (TPA) and cyan‐acrylic acid moieties, for example, have been discovered to be key electron donor and acceptor units, respectively. Various organic groups have been used to modify the electrical and optical properties of light‐sensitive materials. The most well‐known organic compound for achieving such properties is triphenylamine.^[8]^ Idoline.^[9]^ Carbazole^[10]^ and Coumarin^[11]^ have been examined experimentally and theoretically as DSSCS sensitisers. However, it has been reported that metal‐free organic dyes have a power conversion efficiency of up to 13 %,^[12]^ while a portion of the portion of the dye achieves 10 % power conversion efficiency,^[13]^ and metal complexes (Ruthenium‐based dyes) have been reported only 6–9 %.^[14]^ As a result, improving the efficiency of an organic dye is a significant method to boost the PCE of DSSCs. With the emergence of high‐performance computing, computational science emerged as a viable and powerful method of discovering new functional compounds before costly and time‐consuming synthesis.^[15]^ Density functional theory and time‐dependent density functional theory were widely used in the development of DSSCs to investigate the electrical and optical characteristics of a virtual molecules in ground and excited states.^[16]^ In this study, DFT and TD‐DFT have been utilized to examine a series of six d‐π‐A Organic dye molecules illustrated in Figure 1. DFT and TD‐DFT were employed to thoroughly examine these dyes’ electrical structure, optical properties, ICT, electron injection driving force, and light harvesting efficiency, The computed results provide a comprehensive insight of the nature of ICT, as well as critical information for the design and synthesis of D‐π‐A Organic dye for application in DSSCs.[^17^, ^18^] The chemical structures of the dyes under consideration are depicted in Figure 1.

## Computational Details

All computations for the DFT method and TD‐DFT approach were executed using Gaussian 09 software.^[19]^ Which is supported by Gauss view 5.0 for organizing inputs and drawing initial dye structure.^[20]^ The compound‘s geometrical structure was optimized at the electronic ground state by DFT with B_3_LYP hybrid functional.^[21]^ Using 6–31G levels of all atoms (C, H, O, S, N), which have been employed for theoretical analysis of organic dyes for DSSC in both gas phase and solvent.^[22]^ The optimized ground state geometry was utilized to calculate HOMO energy, LUMO energy, and energy gap values, as well as molecular orbital distribution at frontier. The ultraviolent visible absorption spectra were calculated using TD‐DFT/B_3_LYP/6‐31G (d.p) levels, as well as the photophysical properties such as vertical excitation energy, oscillator strength, as well as the contribution of the molecular orbitals responsible for the transition and percentage of composition.^[23]^ The solvent effect was accounted for in the theoretical calculation by employing a polarizable continuum model, the solvent was used to dissolve molecules. When attempting to estimate experimental spectra with reasonable accuracy, the incorporation of solvent influence on the theoretical calculation is critical. Acetonitrile will be employed as the solvent in this study.^[24]^ The calculation in the solvent will be conducted by polarizable continuum model (PCM) in solvent phase,^[25]^ PCM has been used for the study of various chemical and computational calculations of the energy and electronic properties

## 2 Results and Discussions

### 2.1 Optimized Geometry Structure

**Figure 2:**
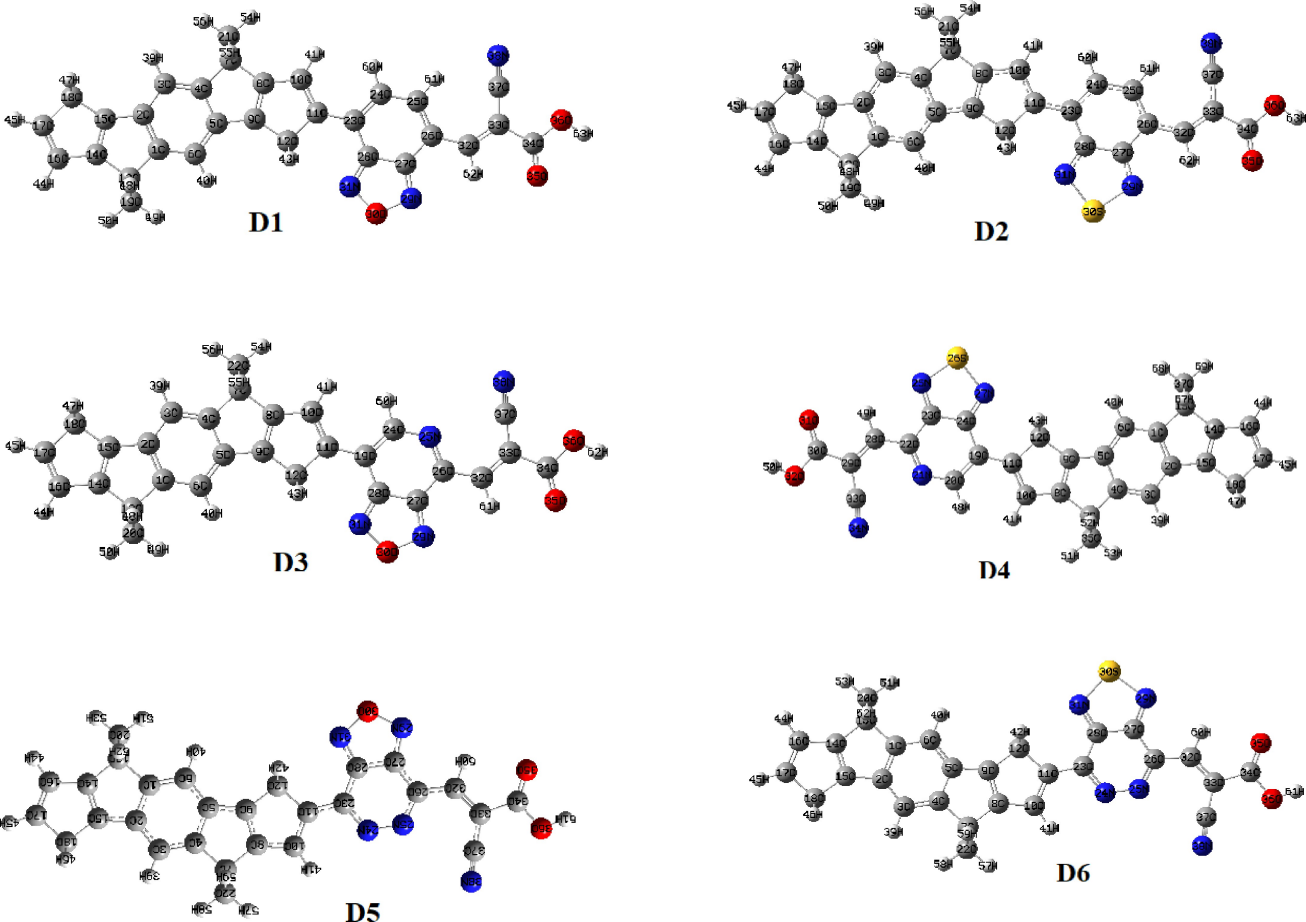
Structural optimization of all studied molecules (D1–D6) in acetonitrile.

Figure 2 depicts the molecular structures and geometrical properties of all dyes investigated. All molecular geometries were estimated at B3LYP functional paired with 6‐31G (d.p) levels using Gaussian 09 software. The geometrical parameters such as bond length and dihedral angle are shown in Tables 1 and 2. Bond lengths between the donor (D) and π‐spacers, as well as the acceptor and π‐spacers, are crucial for serving as intermolecular charge transfer (ICT) bridges. The ICT in the D‐π‐A molecule will benefit from the lower bridging bond length.^[26]^ The computed bond length (Å) of dyes is illustrated in Table 1. The contact between the donor and π‐spacer in the gas phase and acetonitrile solvent is in the range of 1.41608 Å to 1.39669 Å, but the linkage between an electron acceptor and π‐spacer in the gas and solvent phases is in the range of 1.43548 Å to 1.42981 Å. The calculated bond length of each dye investigated and developed in the following sequence between the donor and π‐linker: D5<D6<D3<D1<D4<D3<D2<D4<D1<D5<D2<D6 in gas phase, while the bond length between π‐linker and acceptor increased in the order of D5<D6<D3<D4<D3<D1<D1<D4<D2<D2<D6<D5 in acetonitrile solvent. The results indicate that D5 has a low bond length value. This means that D5 is advantageous for electron transport from the donor to acceptor via π‐spacers. Normally, the degree of conjugation is a critical element influencing dye performance. As a result, the dihedral angle of the examined dyes between the donor part and π‐spacer, as well as between the π‐spacer and acceptor, is collected in Table 2. The dihedral angle is an important characteristic that determines the stability of molecules.^[8]^ The greater the angle of the dihedral of the compound, the higher the steric barrier between the donor and spacers. The acceptor is coplanar with a π‐spacer if the dihedral angle is minimal. If the dye molecules are coplanar, electrons will be transferred from the donor to the acceptor via a π‐conjugated linker. The molecule with the higher dihedral angle will be distorted, and the molecule will become unstable. Dye aggregation will occur as the molecule becomes increasingly twisted. Lower values of dihedral angle mean that the molecule is less distorted and thus more stable. Table 2 shows the dihedral angle rise in gas phases in order of D5<D1<D2<D4<D3<D6, whereas D2<D6<D5<D6<D4<D3<D1 in acetonitrile solvent, According to the data, D5 and D2 have a smaller dihedral angle, and this suggests a lower coplanar effect when compared to other compounds where co‐planarity is detected. As a result, D5 and D2 have smaller dihedral angles in gas and solvent phases because steric barrier between donor and linker is reduced. As a result, these molecules will be in the same planar (coplanar). As a result, the coplanar molecule structure will enhance the electron transport from the donor to acceptor via π‐linker.

**Table 1:** Selected optimized bond length (Å) of the studied dyes (D1–D6).

Dyes	Gas phase		Solvent phase	
	Donor‐π‐spacer (D‐π)	Π‐spacer‐Acceptor (π‐A)	Donor‐π‐spacer (D‐π)	Π‐spacer‐Acceptor (π‐A)
D1	1.43217	1.43508	1.41695	1.41698
D2	1.433699	1.43557	1.42487	1.42171
D3	1.42769	1.43308	1.40837	1.41596
D4	1.43233	1.43407	1.41487	1.41763
D5	1.41608	1.43548	1.39669	1.42981
D6	1.42050	1.43644	1.40302	1.42257

**Table 2:** Selected optimized dihedral angle (Å) of the studied dyes (D1–D6).

Dyes	Gas phase		Solvent phase	
	Angle	Angstrom	Angle	Angstrom
D1	C10−C11−C23−C28	−179.995	C10−C11−C23−C28	179.997
	C33−C32−C26−C27	−179.993	C26−C32−C33−C34	179.999
D2	C12−C11−C23−C24	−179.994	C12−C11−C23−C24	−179.996
	C33−C32−C26−C27	179.989	C33−C32‐26‐V27	−179.997
D3	C12−C11−C19−C28	−179.997	C10−C11−C19−C28	−179.999
	C27−C28−C32−C33	179.997	C33−C32−C26−C27	179.999
D4	C9−C12−C11−C19	−179.991	C12−C11−C19−C20	−179.996
	C29−C28−C22−C23	179.994	C29−C28−C22−C23	179.999
D5	C28−C23−C11−C10	−179.999	C10−C11−C23−C28	−179.995
	C34−C33−C32−C26	−179.993	C27−C26−C32−C33	179.992
D6	C10−C11−C23−C28	179.978	C10−C11−C23−C28	179.993
	C27−C26−C32−C33	179.997	C33−C32−C26−C27	−179.987

### 2.2 Intra Molecular Charge Transfer

Intermolecular charge transfer is an essential object for inorganic sensitizer because it symbolizes the electron flow from donor to acceptor.^[27]^ The measurement utilized to convey charge from an electron source to an electron acceptor is referred to as intra‐molecular charge transfer.^[28]^ When the intermolecular charge transfer rises, the energy levels within HOMO and LUMO stabilize and the energy gap between HOMO and LUMO decreases, resulting in a redshift in the organic photovoltaic spectra^[29]^ If the ICT value is very low, the electron will be transferred directly from the donor to the anchoring group, resulting in charge separation. Impotently, they increase π‐electron delocalization and hence shorten the compound's bond length. The length of bonds connecting the electron donor and π‐spacer is important in the charge transfer from donor to the acceptor in a solar cell since shorter bonds favour the ICT.^[30]^ The greater ICT is usually owing to a nitrogen atom with high electronegativity in the dye ring, which can localize electrons. The donor and π‐linker have positive charges, indicating that these molecules are efficient electron‐pushing units, but the acceptor has negative charges, indicating that it may trap electrons.^[31]^ The intramolecule charge transfer values in the gas phase and acetonitrile solvent are shown in Table 3. ICT advances in order of D5<D1=D6<D3<D4<D2 in gas phases, whereas D5<D3<D6<D1<D4<D2 in acetonitrile solvent. The results demonstrate that D5 has a low ICT value due to its short bonds and strong donor group. As a result, electrons will be transported from the donor group to the acceptor group. As a result, D5 is projected to be a strong candidate for the DSSC application.

**Table 3:** Intramolecular charge transfer of optimized compounds in the gas phase and acetonitrile (in coulomb).

Dyes	In gas	In solvent
D1	5.54×10^−15^	5.53×10^−15^
D2	5.67×10^−15^	5.67×10^−15^
D3	5,46×10^−15^	5.45×10^−15^
D4	5.59×10^−15^	5.58×10^−15^
D5	5.4×10^−15^	5.38×10^−15^
D6	5.54×10^−15^	5.52×10^−15^

### 2.3 Frontier Molecular Orbitals (FMO)

**Figure 3:**
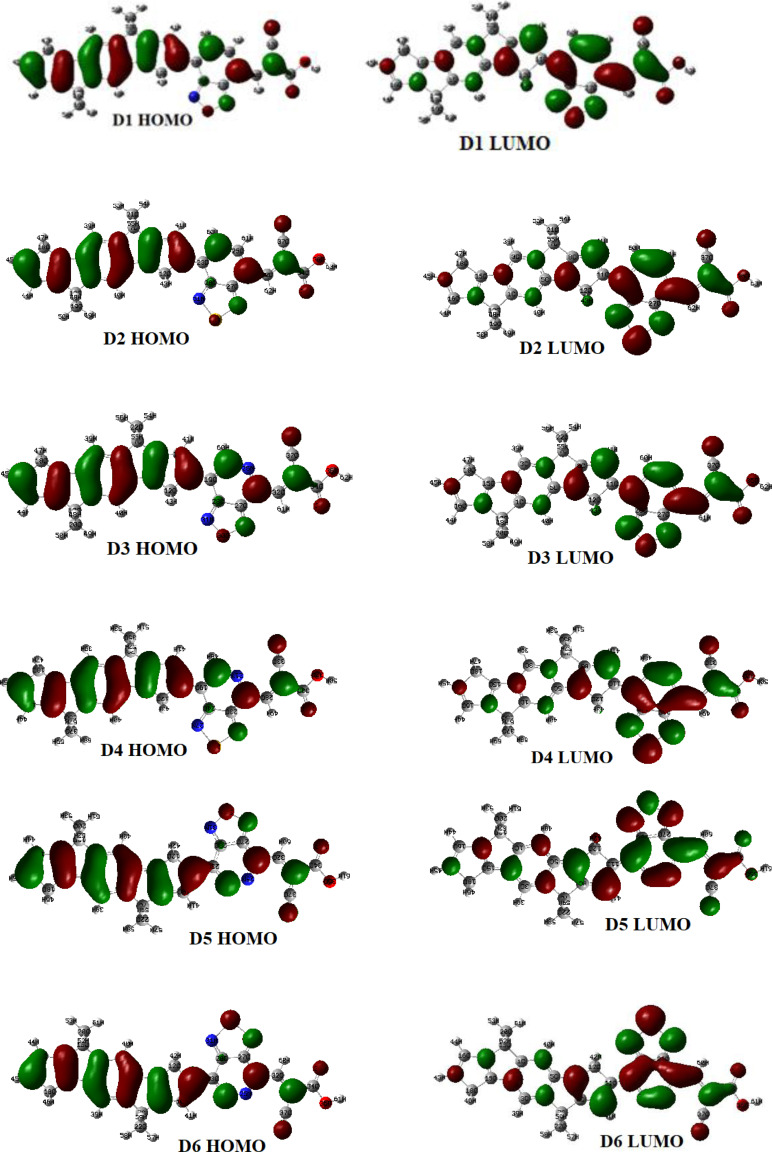
The distributions of f HOMO and LUMO pattern of all studied compounds in acetonitrile.

The contribution of FMO is crucial in determining the ICT and charge‐separated state of the dye molecule.^[32]^ Figure 3 depicts the molecular orbital borders of the dyes investigated. As seen, HOMO electron distributions are concentrated mainly on donor groups and bridges, whereas LUMO electron distributions are primarily localized on the bridge and acceptor units, indicating that HOMOS and LUMOs have good electron‐separated states. Hence, we may anticipate intermolecular charge transfer after the transition happens, which is advantageous for light excitation electron injection.^[33]^ In contrast, HOMO orbitals have bonding quality, whereas LUMO orbitals in all compounds have anti‐bonding properties to achieve the most effective charge separation state.

### 2.4 Electronic Properties

Intermolecular charge transfer from the donor to the acceptor/anchoring group is one of the most important features of organic dye.^[34]^ This property is influenced by the dye's energy, which also influences the photocurrents. Table 4 displays the electronic properties of molecules in gas and solvent phases, such as HOMO energy level, LUMO energy level, energy gap, total energy, and dipole moment of dyes obtained by B3LYP/6‐31G level. Theoretical understanding of HOMO and LUMO energy is essential for studying organic solar cells, such as assessing whether or not the charge transfer between the donor and acceptor is effective.^[35]^ This is critical to note that all dyes have LUMO levels higher than the conduction band, indicating that electron transfer from dyes to the semiconductor will occur.^[36]^ Table 4 shows HOMO energy, LUMO energy, and energy gap for all dyes examined using DFT/B3LYP/6‐31G levels. The calculated HOMO energy levels of dye sensitizers lie in order of D2(−4.924)>D1(−4.999)>D4(−5.016)>D3(−5.103)>D6(−5.202)>D5(−5.295), and the LUMO energies levels lie in the order of D1(−3.454)>D2(−3.380)>D4(−3.623)>D3(−3.692)>D6(−3.715)>D5(−3.780)respectively in acetonitrile solvent. Furthermore, the insertion of π‐conjugated spacer units has a considerable effect on dyes’ HOMO and LUMO energies. Photosensitizers'HOMO and LUMO energy levels are crucial in supplying thermodynamic driving power for electron injection,^[37]^ for separating charges and dye regeneration, the dye's HOMO levels must be significantly more positive than electrolyte's redox potential,^[38]^ while the dye LUMO levels must be more negative compared to the conduction band of semiconductor for injection of electrons from the excited state to conduction band.^[39]^ The findings demonstrated that HOMO energy levels of D1 to D6 dyes are smaller than the value of iodide/tri‐iodide (−4.8 eV) electrolyte to make sure that dyes in the oxidation state can be deoxidized by the electrolyte, whereas LUMO energy levels of D1 to D6 dyes are greater than the edge of conduction band of titanium oxide (−4.0 eV) confirming that electrons in excited state can be transported via the semiconductor's conduction band. A narrower energy gap could increase electron excitation, which improves light harvesting efficiency and has a substantial impact on dye photoelectrical characteristics. The energy gap (Eg) of studied dyes increased in order of D4 (1.393)<D3 (1.411)<D6 (1.487)<D5 (1.517)<D2 (1.544)<D1 (1.545) respectively. The results suggest that D4 has the lowest value of the energy gap in comparison to other dyes. This means that electrons are easily transferred from HOMO to LUMO levels of dye. Table 4 also shows the dyes’ dipole moments. In general, molecules having a larger dipole moment have more asymmetric in their electrical charge distribution. As a result, when exposed to an electric field from the outside, it may become increasingly sensitive and reactive to fluctuations in its electronic framework and electronic features.^[40]^ Table 4 shows that the dipole moment of compounds D3, D4, and D5 is greater than other compounds, implying that these compounds are more reactive and sensitive than other compounds. Indeed, these dyes are preferred for electron liberation. The dye‘s dipole moment rose in the following order: D2 (20.3623)<D1 (22.0049)<D4 (26.1275)<D6 (28.0594)<D2 (8.2775)<D5 (30.1268). The result show that the dipole moments of D3, D5, and D6 dyes are superior to those other dyes, implying that the unique D3, D5, and D6 dyes could be a good choice for boosting the efficiency of the DSSC device.

**Table 4:** Electrical and chemical properties of all studied compounds obtained in acetonitrile (in eV).

solvent phases					
Dyes	HOMO	LUMO	E_T_	Eg	μ(Debye)
D1	−4.999	−3.454	−44186.49	1.545	22.0049
D2	−4.924	−3.380	−52976.29	1.544	20.3623
D3	−5.103	−3.692	−44622.32	1.411	28.2775
D4	−5.016	−3.623	−53412.18	1.393	26.1275
D5	−5.297	−3.780	−45057.44	1.517	30.1268
D6	−5.202	−3.715	−53847.36	1.487	28.0594

### 2.5 Absorption Spectra

**Figure 4:**
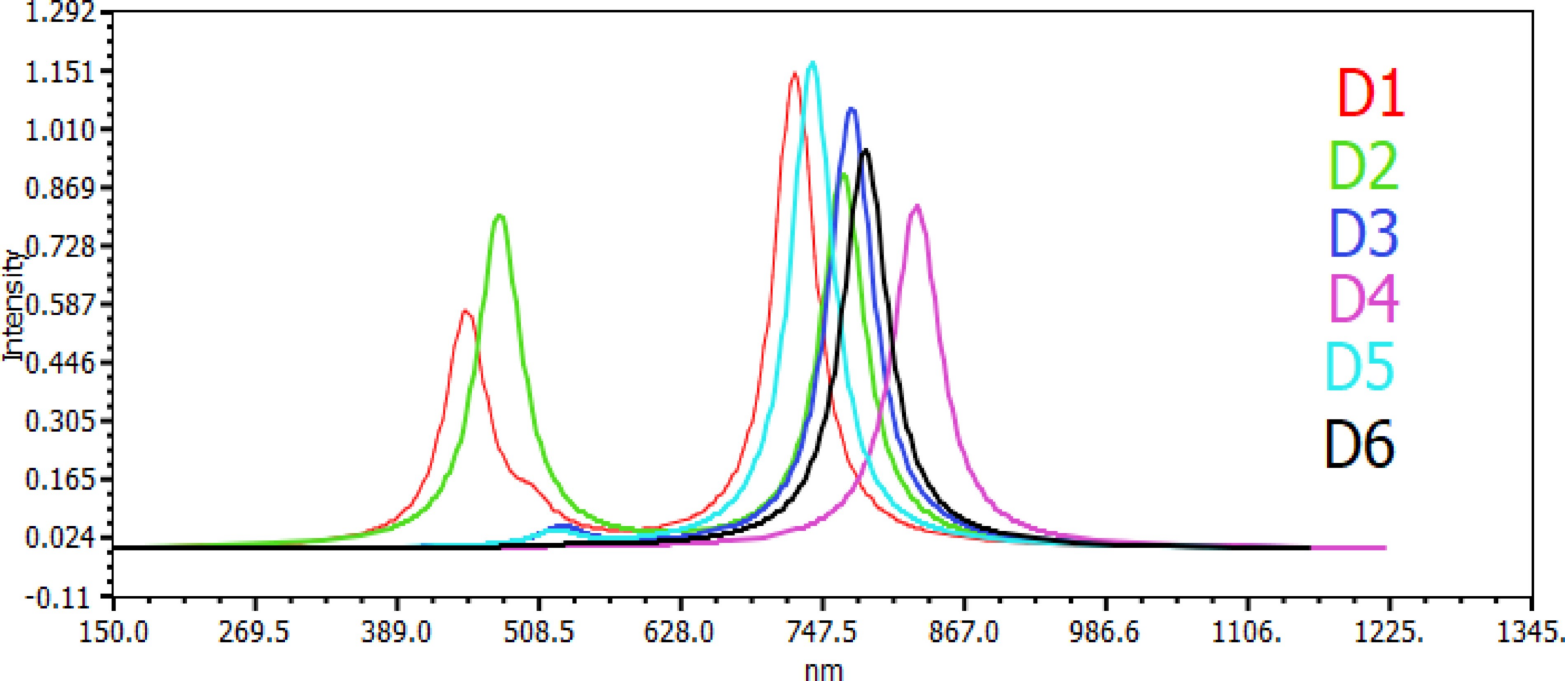
Simulated absorption spectra of all dyes in gas phase.

**Figure 5:**
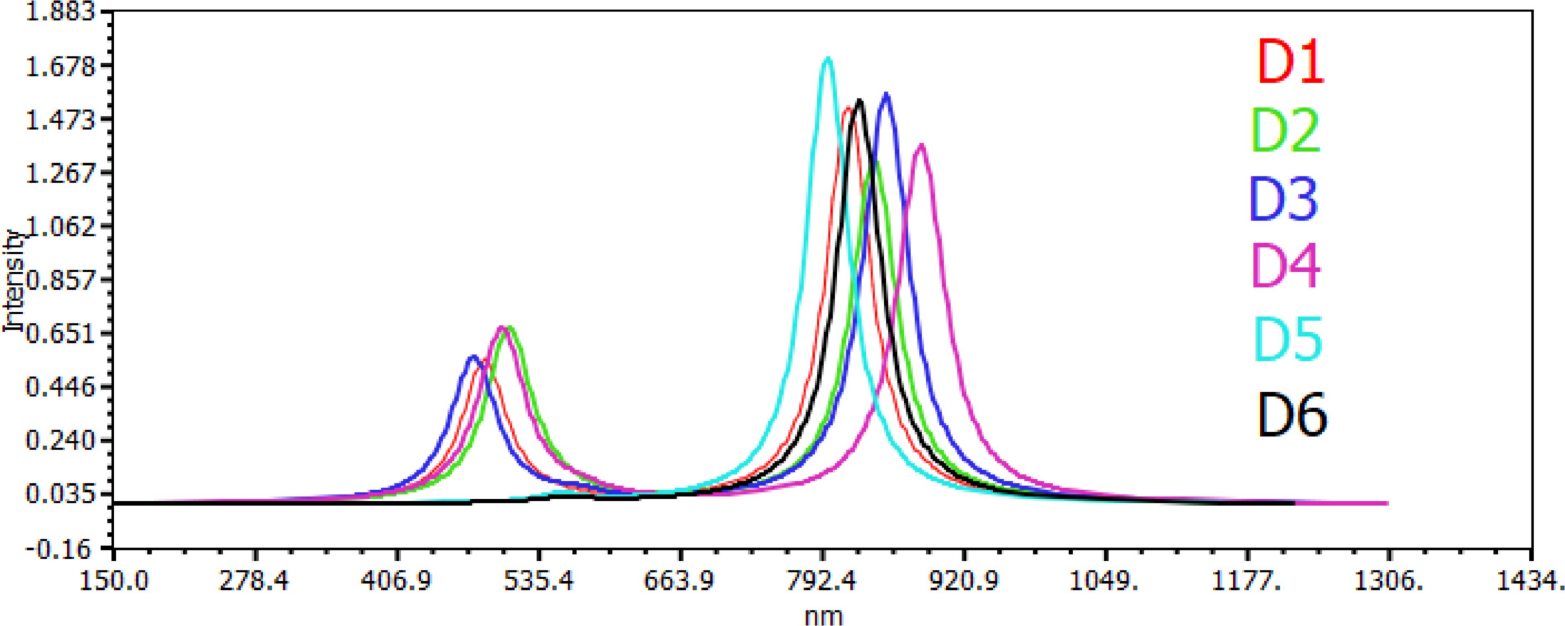
Simulated absorption spectra of all dyes in acetonitrile.

The acknowledgment of the crucial role played by light absorption characteristics in the efficiency of light‐to‐electricity dyes is a well‐established understanding.^[41]^ Dyes with heightened absorption strength and broader band absorption patterns generally exhibit elevated efficiency in this conversion process. Figures 4 and 5 in the study present a detailed visualization of the simulated dye absorption spectra, specifically conducted in acetonitrile solvent and obtained through the TD‐DFT/B3LYP/6‐31G levels. Tables 5 and 6 complement the visual representation by providing a comprehensive breakdown of essential optical parameters. These include the projected maximum absorption wavelengths, oscillator strength, transition energy, and vertical excitation energies in both the gas phase and acetonitrile.

**Table 5:** Calculated absorption spectral data of the studied organic dye molecules in the gas phase.

Gas					
Dyes	SN	λ_max_ (nm)	ΔE (eV)	(f)	MO Contribution
D1	S1	724.92	1.7103	1.1468	HOMO→LUMO+1 (100 %)
					HOMO‐1→LUMO+1 (3.35 %)
	S2	503.72	2.4614	0.0673	HOMO→LUMO+2 (88.46 %)
					HOMO→LUMO+3 (9.11 %)
	S3	447.96	2.7678	0.5613	HOMO→LUMO+2 (93.96 %)
					HOMO→LUMO+3 (2.19 %)
D2	S2	765.58	1.6195	0.8997	HOMO→LUMO+1 (100 %)
					HOMO→LUMO+2 (2.05 %)
					HOMO‐1→LUMO (2.96 %)
	S2	518.60	2.3907	0.0024	HOMO→LUMO+1 (87.50 %)
					HOMO→LUMO+2 (4.99 %)
					HOMO→LUMO+3 (6.21 %)
	S3	476.21	2.6036	0.7976	HOMO→LUMO+2 (4.24 %)
					HOMO→LUMO+1 (2.48 %)
					HOMO→LUMO+2 (91.05 %)
D3	S1	772.48	1.6050	1.0630	HOMO→LUMO+1 (100 %)
					HOMO‐1→LUMO (4.53 %)
	S2	529.09	2.3433	0.0466	HOMO→LUMO+2 (90.79 %)
					HOMO→LUMO+3 (8.47 %)
	S3	465.91	2.6611	0.0003	HOMO→LUMO+4 (97.82 %)
D4	S1	827.54	1.4982	0.8252	HOMO→LUMO+1 (100 %)
					HOMO‐1→LUMO (4.12 %)
	S2	546.93	2.2669	0.0022	HOMO→LUMO+2 (91.98 %)
					HOMO→LUMO+3 (6.05 %)
	S3	484.62	2.5584	0.0002	HOMO→LUMO+4 (98.44 %)
D5	S1	739.63	1.6763	1.1744	HOMO→LUMO+1 (100 %)
					HOMO‐1→LUMO (4.62 %)
	S2	681.13	1.8203	0.0000	HOMO→LUMO+3 (99.20 %)
	S3	523.02	2.3705	0.0347	HOMO→LUMO+2 (89.08 %)
					HOMO→LUMO+3 (10.05 %)
D6	S1	784.22	1.5810	0.9605	HOMO→LUMO+1 (100 %)
					HOMO‐1→LUMO (4.39 %)
	S2	717.91	1.7270	0.0000	HOMO→LUMO+3 (99.34 %)
	S3	538.56	2.3021	0.0044	HOMO→LUMO+2 (91.56 %)
					HOMO→LUMO+3 (7.25 %)

**Table 6:** Calculated absorption spectral data of studied organic dye molecules in acetonitrile.

Solvent phases					
Dyes	SN	λ_max_ (nm)	ΔE (eV)	(f)	MO Contribution
D1	S1	816.87	1.5178	1.5216	HOMO→LUMO+1 (100 %)
					HOMO‐1→LUMO (3.91 %)
	S2	543.45	2.2814	0.0046	HOMO→LUMO+2 (90.57 %)
					HOMO→LUMO+2 (2.79 %)
					HOMO→LUMO+3 (5.95 %)
	S3	487.13	2.5452	0.5471	HOMO→LUMO+2 (92.50 %)
					HOMO→LUMO+3 (3.73 %)
D2	S2	840.21	1.4756	1.3148	HOMO→LUMO+1 (100 %)
					HOMO‐1→LUMO(3.40 %)
	S2	549.09	2.2580	0.0198	HOMO→LUMO+2 (87.65 %)
					HOMO→LUMO+2 (6.81 %)
					HOMO→LUMO+3 (4.29 %)
	S3	509.17	2.4350	0.6651	HOMO→LUMO+2 (5.70 %)
					HOMO→LUMO+2 (89.45 %)
					HOMO→LUMO+3 (3.09 %)
D3	S1	850.83	1.4572	1.5666	HOMO→LUMO+1 (100 %)
					HOMO‐1→LUMO (5.90 %)
	S2	569.86	2.1757	0.0265	HOMO→LUMO+2 (91.57 %)
					HOMO→LUMO+3 (7.92 %)
	S3	477.22	2.5981	0.5578	HOMO→LUMO+2 (94.71 %)
D4	S1	882.75	1.4045	1.3699	HOMO→LUMO+1 (100 %)
					HOMO‐1→LUMO (5.41 %)
	S2	576.26	2.1515	0.0275	HOMO→LUMO+2 (92.40 %)
					HOMO→LUMO+3 (6.33 %)
	S3	502.65	2.4666	0.6662	HOMO→LUMO+2 (94.93 %)
D5	S1	797.87	1.5539	1.7123	HOMO→LUMO+1 (100 %)
					HOMO‐1→LUMO (5.16 %)
	S2	615.34	2.0149	0.0002	HOMO→LUMO+3 (98.66 %)
	S3	556.16	2.2293	0.0298	HOMO→LUMO+2 (86.60 %)
					HOMO→LUMO+2 (2.88 %)
					HOMO→LUMO+3 (9.59 %)
D6	S1	826.36	1.5004	1.5508	HOMO→LUMO+1 (100 %)
					HOMO‐1→LUMO (4.94 %)
	S2	628.74	1.9719	0.0001	HOMO→LUMO+3 (98.88 %)
	S3	560.78	2.2109	0.0203	HOMO→LUMO+2 (88.24 %)
					HOMO→LUMO+3 (9.53 %)

The vertical excitation energy progression is elucidated, indicating an order in the gas phase as D6<D5<D4<D3<D2<D1. In the solvent (acetonitrile), the order is D6<D5<D4<D2<D1<D3. Notably, all dyes exhibit higher vertical excitation energy in the gas phase compared to their acetonitrile solution counterparts. The relevance of vertical excitation energy in the study is underscored by its predictive capability regarding excited state features in larger or medium‐sized molecules. Additionally, it serves as a metric for evaluating how the energy difference varies between different chemical systems, providing valuable insights into the performance of the TD‐DFT excitation energy.^[42]^

The divergence in transition character contributions between the gas phase and solvent (acetonitrile) stems from the impact of the polar environment on electronic energy, as elucidated in the study.^[43]^ This environmental difference leads to distinctive patterns in the contributions of dye molecules to transitions in both gas and acetonitrile phases.In the gas phases, the contributions of dye molecules exhibit an increasing order: D1<D4<D3<D6<D5. This sequence suggests a hierarchy in the transition contributions of these molecules within the gas environment. Conversely, in acetonitrile solution, the order is D2<D1<D4<D3<D6<D5.

Notably, D4 and D5 emerge as the dye molecules with the largest contribution to transitions in both the gas phase and acetonitrile solution. The prominence of D4 in transition contributions indicates its suitability for applications in dye‐sensitized solar cells (DSSCs). Transition strength, measured through oscillator strength, is crucial for evaluating the efficiency of light harvesting. The calculated oscillator strength for the dyes in gas phases follows an order of D1>D5>D3>D6>D2>D4. In acetonitrile solution, the order is D1>D4>D2>D5>D3>D6.

Significantly, the study reveals that the oscillator values in the gas phase differ from those in the solvent solution due to the addition of acetonitrile solution to the organic dye molecule. Consequently, D1 compounds exhibit higher oscillator strengths in both gas phases and acetonitrile solution compared to other compounds, indicating superior performance and a potential for higher efficiency in light harvesting applications. This underscores the significance of D1 in achieving enhanced light‐to‐electricity conversion efficiency in the context of dye‐sensitized solar cells. The study examines the optimal absorption wavelengths of all organic dye molecules in both gas phase and solvent (acetonitrile), providing valuable insights into their light absorption characteristics. In the gas phase, the order of optimal absorption wavelengths is listed as D6>D3>D2>D5>D1, while in acetonitrile solution, the order is D4>D3>D2>D6>D1>D5.

The concept of redshift is introduced, wherein larger wavelength molecules exhibit a redshift in their absorption spectra. This shift towards longer wavelengths is known to impact the photocurrent conversion efficiency of dye‐sensitized solar cells (DSSCs). In particular, the study highlights D5 as having higher maximum absorption peaks falling within the substantial range of 680 to 860 nm. This specific range is crucial for efficient sunlight absorption, aligning with the solar spectrum. Furthermore, the study emphasizes the dependence of maximum absorption peaks on the Highest Occupied Molecular Orbital (HOMO) energy levels. This dependency indicates a correlation with the efficiency of the electrolyte in the context of DSSCs. As the HOMO orbital energy levels play a role in determining the absorption capacity, their influence on the maximum absorption peaks suggests a connection between the molecular properties of the dyes and the overall performance of the electrolyte. This finding provides valuable insights into the factors influencing the efficiency of dye‐sensitized solar cells and underscores the importance of considering both molecular and environmental factors in optimizing these systems for enhanced light absorption and energy conversion.

As shown in Figures 4 and 5. We notice that the dye has a higher maximum absorption peak, which helps enhance the injection efficiency than other compounds, meaning that this molecule could collect more sunlight at a longer wavelength, which is a valuable property for DSSC production as it increases the photovoltaic conversion efficiency., and can. Therefore, be qualified as a significant candidate for the application of DSSC. This result is in excellent agreement with the calculated energy gas (E_gap_) value for D4‐designed dye.

### 2.6 Photovoltaic Parameters

The power conversion efficiency (PCE) of solar cells is the most commonly used parameter for evaluating the performance of various solar cells, it is proportional to the open‐circuit voltage, as shown in the expression below.^[44]^

(1) $$\eta =\frac{Jsc\;Voc\;FF\;}{Pinc}\;$$

Where J_SC_ is the device's DSSC short‐circuit current density when no voltages are applied, FF is the fill factor, V_OC_ is the maximum open‐circuit voltage, and P_inc_ is the constant incident sunlight energy. Open‐circuit is one of the metrics used to evaluate the performance of a solar cell; it is obtained at zero‐level current indicating the voltage generated from a solar device. To achieve the maximum PCE, the VOC must be as large as possible, it can be calculated by subtracting the acceptor's LUMO energy from the donor's homo energy within the solar cell. Using Eq (2), the theoretical value may be calculated.
(2)

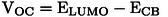




ELUMO indicated the energy level associated with dyeing its excited state, while E_CB_ denoted the energy of the TiO_2_ conduction band (−4.0 eV). Open‐circuit voltage is the most important aspect in determining external maximum voltage units for DSSCs. J_SC_ is represented by meanslight harvesting efficiency ability, and electron injection free energy (ΔG^inject^), J_SC_ is written as follows.^[7]^

(3)

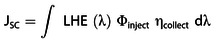




Where Φ^inject^ denotes the electron injection efficiency, η^collect^indicate charge collection efficiency, LHE (λ) denotes the light gathering efficiency at a specific wavelength. Higher LHE values are helpful for enhancing the current JSC and hence the incoming photon to electron conversion efficiency, and LHE may be computed using eq 4.^[45]^

(4)

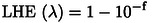




Where f denotes the oscillator's strength proportional to the highest dye concentration. It has been discovered that the greater the oscillator strength value, the higher the LHE for sensitizers and, thus, the higher the value of J_SC_. This power has an immediate effect on LHE. The free energy of an electron injection can be expressed using Eq 5, ^[23]^

(5) $${\Delta G}^{\mathrm{inject}}=E^{{\mathrm{dye}}^{*}}-E_{\mathrm{CB}}$$

Where ΔG^injec^t signifies the amount of free energy change for electron injection efficiency, E^dye*^denotes the excited molecule‘s oxidation potential energy, and ECB denotes the TiO_2_ conduction band's reduction potential (E_CB_=−4.0 eV). The following formula can be used to calculate the value of 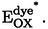 6, ^[46]^

(6)

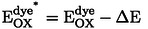




Where $E_{OX}^{dye}$ represents oxidation potential energy of the dye in its ground state, and ΔE is the vertical excitation energy equivalent to the maximal absorption (λ_max_). Furthermore, the underlying force of electron regeneration (ΔG^reen^) having the smallest value indicates the fastest electron process, and it can be determined using Eq. 7., ^[47]^

(7)

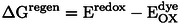




Where E^redox^denotes electrolyte iodide/tri‐iodide (−4.8 eV) redox potential.

Injection driving power (ΔG^inject^), light harvesting efficiency, and open‐circuit voltage value were computed using formulae and compiled in Table 7. The potential energy of ground ($E_{OX}^{dye}$ ), and excited oxidation state ( 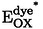 ), electron for excited oxidation state (E^dye^*), the smaller the excited oxidation state is, the easier it is for the DSSCs to generate light excitation, which is the initial stage of DSSC operation. From Table 7, D5 has a smaller Eye^*^ value (−6.8509 eV in acetonitrile solvent) than other dyes, indicating that D5 is more susceptible to photoexcitation. From Table 7, all the calculated ΔG^inject^ values of dye are negative, which indicates that the conduction band edge of TiO_2_ lies below the excited state of dye[^48]^ and thus favours electron injection. ΔG^regen^ is a term used to describe the ability of the dye to regenerate from the electrolyte. The driving force of regeneration (ΔG^regen^) can be used to describe the regeneration efficiency. Rapid regeneration of oxidized dye aids in the improvement of DSSC efficiency. As shown in Table 7, the ΔGregen values of the design dye are less than those of the other dyes, meaning that the design dyes can be easily regenerated and result in improved power conversion efficiency. It is also clear from Eq.4 that the LHE and ΔG^inject^ are critical parameters influencing J_SC_; dyes with higher LHE values can absorb more sunlight and convert it into energy. From Table 7, LHE values increased in the order of D2<D4<D1<D6<D3<D5, we observe that all dyes investigated have a greater LHE value than other dyes, indicating that these dyes have a high absorption ability. The open‐circuit voltages (V_OC_) are another element that determines the power conversion efficiency of dyes. A higher V_OC_ value in a molecular dye indicates greater electron injection power. From Table 7, the highest values are found in D1 and D2, with values of 0.546 eV and 0.620 eV, respectively, and the trend is as follows D2>D1>D4>D3>D6>D5.

**Table 7:** Calculated photovoltaic properties of the studied dyes (in eV).

Acetonitrile Solvent						
Dye	$E_{OX}^{dye}$	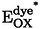	ΔG^inject^	ΔG^regen^	LHE	VOC
D1	−4.999	−6.5168	−2.5168	0.199	0.9699	0.546
D2	−4.924	−6.3996	−2.3996	0.124	0.9516	0.620
D3	−5.103	−6.5602	−2.5602	0.303	0.9729	0.308
D4	−5.016	−6.4205	−2.4205	0.216	0.9573	0.377
D5	−5.297	−6.8509	−2.8509	0.497	0.9806	0.220
D6	−5.202	−6.7024	−2.7024	0.402	0.9719	0.285

## 3 Conclusions

In conclusion, this comprehensive theoretical study delved into the geometry and electrical properties of six D‐π‐A organic dye molecules (D1‐D6) for potential application in dye‐sensitized solar cells (DSSCs). Utilizing DFT and TD‐DFT methods, the investigation focused on electronic structure, absorption spectra, and photovoltaic properties. The results highlight the suitability of D1‐D6 compounds as DSSCs sensitizers, evidenced by their favorable LUMO energies surpassing the TiO2 conduction band and lower HOMO energies relative to the electrolyte‘s redox potential. Particularly, the D4 dye stands out with a narrower energy gap and a distinctive red‐shift absorption spectrum, indicating its potential for enhanced performance. The adjustable donor group in D4 emerges as a promising functional component for the D‐π‐A structure, offering valuable insights for future experimentation and the application of D4 in highly efficient DSSCs.

## Conflict of interests

The authors declare no conflict of interest.

4

## Data Availability

The data that support the findings of this study are available on request from the corresponding author. The data are not publicly available due to privacy or ethical restrictions.
